# SCMeTA: a pipeline for single-cell metabolic analysis data processing

**DOI:** 10.1093/bioinformatics/btae545

**Published:** 2024-09-06

**Authors:** Xingyu Pan, Siyuan Pan, Murong Du, Jinlei Yang, Huan Yao, Xinrong Zhang, Sichun Zhang

**Affiliations:** Department of Chemistry, Tsinghua University, Beijing 100084, China; Department of Chemistry, Tsinghua University, Beijing 100084, China; Department of Chemistry, Tsinghua University, Beijing 100084, China; Department of Chemistry, Tsinghua University, Beijing 100084, China; Division of Chemical Metrology and Analytical Science, National Institute of Metrology China, Beijing 100029, China; Department of Chemistry, Tsinghua University, Beijing 100084, China; Department of Chemistry, Tsinghua University, Beijing 100084, China

## Abstract

**Summary:**

To address the challenges in single-cell metabolomics (SCM) research, we have developed an open-source Python-based modular library, named SCMeTA, for SCM data processing. We designed standardized pipeline and inter-container communication format and have developed modular components to adapt to the diverse needs of SCM studies. The validation was carried out on multiple SCM experiment data. The results demonstrated significant improvements in batch effects, accuracy of results, metabolic extraction rate, cell matching rate, as well as processing speed. This library is of great significance in advancing the practical application of SCM analysis and makes a foundation for wide-scale adoption in biological studies.

**Availability and implementation:**

SCMeTA is freely available on https://github.com/SCMeTA/SCMeTA and https://doi.org/10.5281/zenodo.13569643.

## 1 Introduction

Metabolites within the cells encapsulate every cellular life activities, and these molecules disseminate critical life information (Zhang *et al.* 2013), thereby significantly contributing to our understanding of life processes and disease mechanisms (Ali *et al.* 2019, Gomollón-Bel 2021). Owing to the extremely small volume and complex contents of a mammalian cell, MS is therefore the method of choice for single-cell metabolism (SCM) analysis because of its high sensitivity and the ability to identify metabolites by structure elucidation (Zhu *et al.* 2018, Cheng *et al.* 2022, Notarangelo *et al.* 2022). In the past decades, a variety of methods have been developed, which have driven the advancement of single-cell metabolic analysis (Masujima 2009, Fujii *et al.* 2015, Yao *et al.* 2019, Seydel 2021). Those research requires extensive data processing, including extracting a large number of mass-to-charge ratio features and their abundances, making the handling of single-cell metabolic data extremely complex and challenging (Ali *et al.* 2019). There are already numerous tools and software available for data processing in proteomics and transcriptomics (Amezquita *et al.* 2019, Zhou and Troyanskaya 2021, Gatto *et al.* 2023). However, the data processing for SCM analysis still lacks a unified workflow and standardized software (Zhu *et al.* 2021, Zhang *et al.* 2023), resulting in insufficient interoperability between different methods. Therefore, it is essential to establish a transparent and efficient processing workflow to connect the original data with biological interpretation.

To support rigor and reproducibility in single-cell metabolism research (Supplementary Fig. S1), we have developed a processing workflow for time-series-based single-cell metabolic data analysis named SCMeTA. It retains an extensible interface and plugin system for adapting to the data from various instruments. We conduct analysis on single-cell data acquired from QE-Orbitrap MS, while preserving the extensibility through Application Programming Interfaces (APIs) and plug-ins to accommodate the data of other instruments. The SCMeTA library incorporates modules for data import, pre-processing, single-cell data screening, metabolite screening, and visualization, each specifically optimized for single-cell metabolic data. SCMeTA has significant practical value in improving the application of single-cell metabolic analysis, and it also lays the foundation for future research on single-cell metabolomics on a larger scale. To assist users to better utilize SCMeTA, we provide online documentation at https://sc-meta.com, which offers detailed introduction about the installation, usage, and component extension development of SCMeTA.

**Figure 1.**
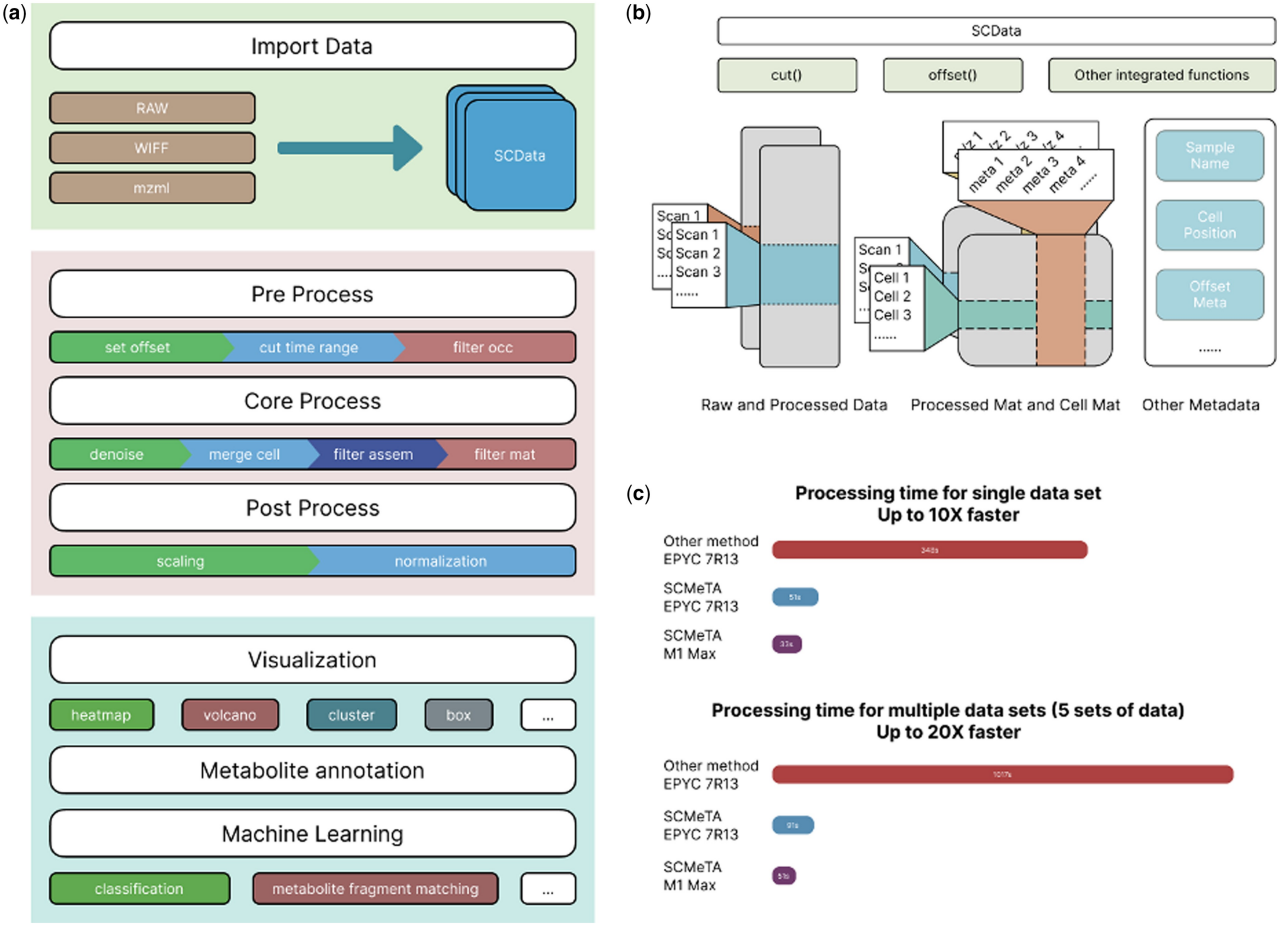
(a) The SCMeTA pipeline processes raw mass spectrometry data into SCData instances. This process includes offset setting, data trimming, core-mass ratio filtering, denoising, cell merging, signal-to-noise ratio screening, and cell matrix filtering. The resulting matrix undergoes log transformation and standardization and can be used for further analysis like visualization and machine learning. (b) The SCData class stores SCM profiling data for a time course. It includes methods for data manipulation and stores both raw and preprocessed mass spectrometry data, with scan frames and mass-to-charge ratio info. The core data processing is represented as a 2D matrix with columns and rows representing metabolic and cell information respectively. It also stores other sample-related information. (c) Speed comparison between SCMeTA and traditional method in MATLAB.

SCMeTA provides a highly mutually dependent data management approach. It is developed using the object-oriented programming language Python, with optimization encapsulation carried out in various functions, achieving modular and scalable software development. The library offers the ability to handle single-cell data generated by different mass spectrometry manufacturers on various platforms (Linux/macOS/Windows), with the capacity to directly import Thermo RAW, Waters WIFF, as well as other formats. The SCMeTA processing method, built on the numpy and pandas libraries, significantly boosts the speed of data processing. Compared to the MATLAB-based method (Yao *et al.* 2019), SCMeTA achieves up to 20 times the processing speed (Fig. 1c). Meanwhile, SCMeTA can also be invoked in MATLAB, Docker containers or directly in the Jupyter Notebook in a web page. Once processing is completed, SCMeTA also provides a series of downstream analysis tools and can export common analyzable matrix data of metabolic in single cell.

## 2 Features and methods

SCMeTA offers an integrated and standardized workflow that is flexible and compatible, capable of handling data from single cells or high-throughput single-cell groups. The step-by-step data analysis process is primarily depicted in Fig. 1a.

### 2.1 Data import

SCMeTA accommodates the diversity of single-cell metabolism detection methods and vendor data formats by providing various data importation strategies, including clustering methods for data distributed over multiple files and centralized methods for storing numerous cells within a single file. By using a Python to .NET integration library, SCMeTA enables rapid data import across different operating systems (Windows/macOS/Linux) and from multiple instrument manufacturers, including Thermo, Waters, and other formats. Cells data is stored into a comprehensive DataFrame within the special-designed data container called SCData.

### 2.2 Data container

SCData is for storing single-cell metabolism data and raw data in SCMeTA (Fig. 1b). SCData contains raw data and preprocessed data stored in the form of a multi-column DataFrame, including parsed cell retention time (scan positions) and single-cell metabolism matrix: where rows represent metabolic features and columns represent cells. SCData also includes a series of preprocessing methods, including mass spectrometry data offset correction, data segmentation, etc.

### 2.3 Preprocessing

The data gleaned from single-cell samples tends to be immensely precious. To augment the utilization of single-cell data, a spectrum of preprocessing techniques for the imported raw data is provided, including data sectioning (“cut”) and spectral drift (“offset”) as corrective measures. These procedures enable effective extraction of cell data within specified timeframes, as well as adjustment of spectral quality axis deviations.

In the mass spectrometric analysis, resolution is a critical parameter to evaluate the performance of analytical instruments, affecting whether we can accurately determine the composition of metabolites. To maintain a credible detection resolution of mass spectrometry, it is essential to implement a data processing function known as “filter occurrences.” This function consolidates mass-to-charge (*m*/*z*) ratios by merging adjacent peaks within the threshold of reliable analytical resolution. The process involves the aggregation of all *m*/*z* values and their corresponding ion intensities based on predefined mass intervals, effectively streamlining scattered data points, and minimizing signal redundancy caused by overlapping peaks. We usually use a resolution of 0.01 to match high-resolution mass spectrometers such as Orbitrap QE and filter out signal peaks that occur <10 times. Consequently, the consolidated dataset resulting from this integration more clearly reflects the true metabolite profile of the sample and aligns with the instrument's inherent high-resolution capabilities (Supplementary Fig. S7).

### 2.4 Core processing

#### 2.4.1 Noise reduction

Due to the continuous fluctuations of small molecule metabolites in biological activities, different methods may lead to deviations in the measurement results noise when measuring single-cell metabolite data, which often detrimentally impacts cell detection results (Supplementary Fig. S5). Conventional noise subtraction methods could significantly skew the accuracy for single cells. Therefore, we have developed a unique noise extraction algorithm specifically for single-cell data, which distinctly analyze noise around each cell rather than using total noise as the cells’ matched noise to better restore the metabolite information of the single cell. Firstly, we extract the list of valid detection information in cells through a three-times signal-to-noise ratio method, then carry out specific noise subtraction for each cell in the data (Supplementary Fig. S2).

#### 2.4.2 Metabolite filtration

The typical readout of metabolomics based on mass spectrometry measurement is a large matrix encompassing the detected mass-to-charge ratios (*m*/*z*) signatures along with their abundances. Yet, a large quantity of the data is often inundated with nonsignificant peaks while parsing the mass spectrum of mass-to-charge ratios. To efficiently identify and interpret the singular cell characteristic metabolites, we have conceived a metabolite filtration functionality base on the frequency of metabolites appearing in all cells. This feature executes filtration on a substantial number (surpassing 10 000) of mass-to-charge signals depending on the number of cells and the frequency of occurrence of the mass spectrometry signals, therefore yield reliable metabolites that more accurately reflect the status of the examined cells. In our function, setting the threshold to 10%–20% can more effectively filter out background signals and noise peaks (Supplementary Figs S10 and S11).

#### 2.4.3 Normalization and standardization

The principal aim of normalization is to minimize the measurement variations across samples to the utmost extent, to confer consistency and comparability among discrete SCM data. Pertaining to the predisposition of single-cell data to be measured in batches, tempering batch effect disruption is required to maintain data coherence and reliability. Thus, we afford an array of commonplace normalization methods available for invocation during the normalization course. Choosing the appropriate normalization method effectively mitigates interspersed batch effect, laying a solid foundation for the subsequent data analysis reliability.

### 2.5 Downstream statistical analysis

A visualization module based on Matplotlib for SCM analysis is including in SCMeTA. This kind of visual presentation, especially in dimension reduction, is effective in communicating and interpreting results particularly in handling complex bio-data. SCMeTA has integrated dimension reduction visualization for cell data, including methods like Kernel-PCA, t-SNE, UMAP which show excellent dimension reduction results for nonlinear data. The visualization module also comprises a suite of modules for single-cell intra-variability metabolite analysis, like heat maps, volcano plots, and box plots. These graphical features facilitate quick and efficient identification of characteristic metabolic data within the experimental group.

Peak identification of SCM data constitutes a key step in single-cell metabolomics and forms the foundation for metabolomic research. The accuracy of peak identification directly impacts the quality of subsequent data analysis. SCMeTA has an inbuilt local HMDB metabolite identification system which quickly and efficiently ascertains accurate mass number corresponding to metabolite information for primary mass spectra.

## 3 Case study

SCMeTA was validated through the analysis of elongated cell signals using automated single-cell analysis technology (Chen *et al.* 2022) and high-throughput metabolite detection in flow cytometry (Yao *et al.* 2019), we designed three different experiments to verify the performance of SCMeTA (Supplementary Experiments S1–S3). The results show that SCMeTA has excellent noise removal effect in single-cell metabolism detection (Supplementary Fig. S7) and significant preservation of over 600 cell metabolite peaks. SCMeTA *proved* efficient in processing multi-cell data (Fig. 1c), discerning metabolic differences between cell types (Including cancer cells and lymphocytes in actual blood.) with clear cluster analysis results and demonstrating consistency within cells of the same type (Supplementary Fig. S9), highlighting its effectiveness in reducing batch effects in single-cell metabolite experiments (Supplementary Fig. S8).

## Supplementary Material

btae545_Supplementary_Data

## Data Availability

The data underlying this article will be shared on reasonable request to the corresponding author.

## 4 Conclusion

SCMeTA is a python library developed specifically for single-cell metabolomics data, used for rapid processing of single-cell metabolomics mass spectrometry data and metabolite data analysis. This library enables the possibility of processing large-scale single-cell metabolomics data. SCMeTA is flexible, adaptable to various single-cell metabolomics research methods, and can be further expanded through plugin integration.
