# Supplementary material for: SCMeTA: a pipeline for single-cell metabolic analysis data processing
Source: Bioinformatics. 2024 Sep 6;40(9):btae545. doi: 10.1093/bioinformatics/btae545 (PMC11401741; doi:10.1093/bioinformatics/btae545)
Supplement: btae545_Supplementary_Data [file btae545_supplementary_data.docx]

**Supporting Information**

**Experiment S1**

Collected single-cell data via CyESI from cross experiments, with daily experiments conducted alternately between HeLa and HepG2 cells for two days. Each group collected approximately 30 minutes of mass spectrometry positive mode data. Use SCMeTA to process multiple sets of data simultaneously and analyze the differences and batch effects between multiple sets of data using the t-SNE dimensionality reduction method.

**Experiment S2**

Use droplet-extraction to extract contents from individual HeLa cells and detect them using pico-ESI-MS technology. The signals of the extracted cellular components were processed with SCMeTA for analysis, obtaining the pre-noise deduction signal and post-processing signal of the cellular extracts.

**Experiment S3**

Collect peripheral blood mononuclear cells (PBMCs) from human subjects, use flow cytometry to sort specific subsets of four cell types including monocytes, natural killer (NK) cells, T cells, and B cells. Then collect their mass spectrometry data using CyESI-MS and analyze data with SCMeTA.

**Figure S1**


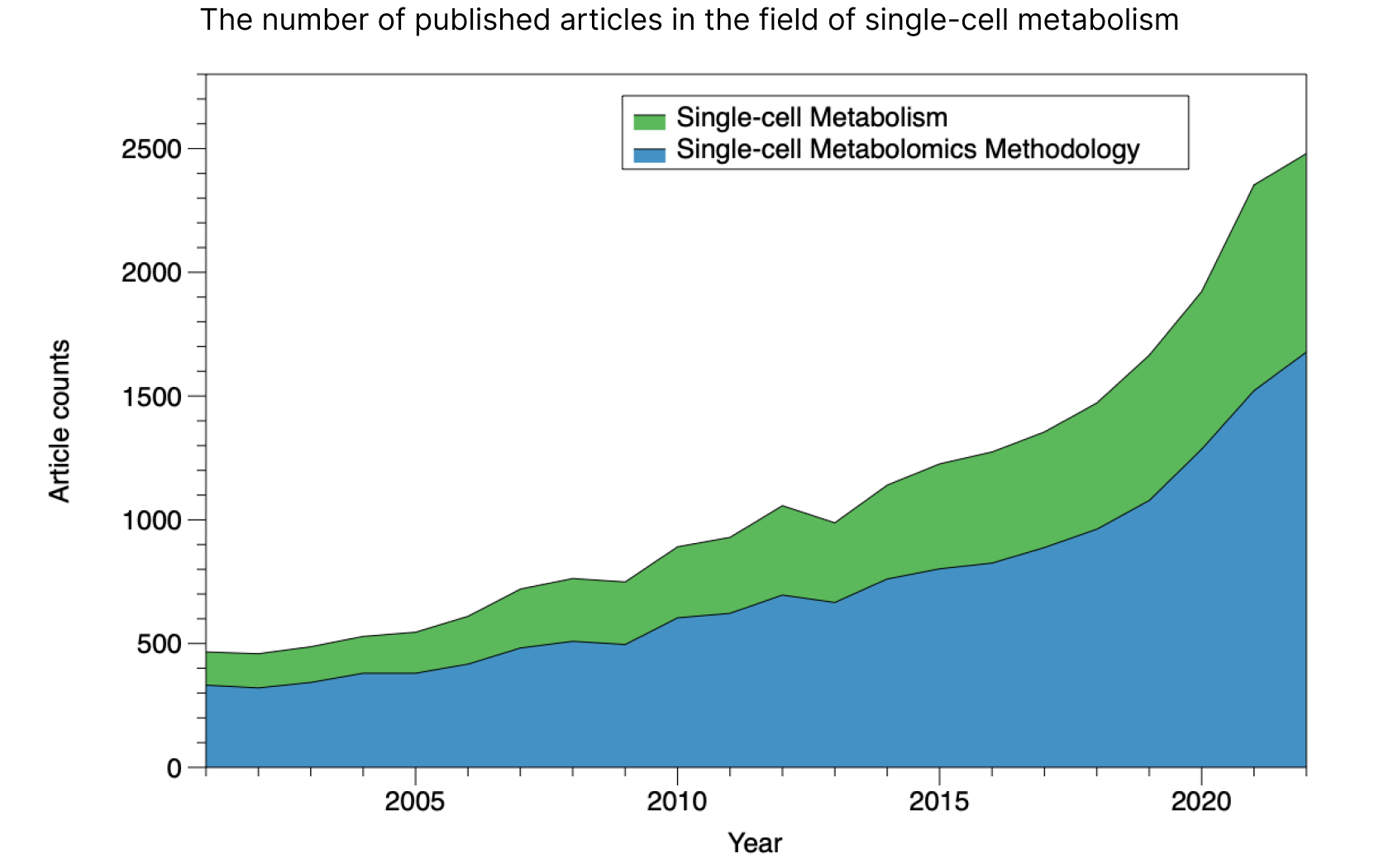


Figure S1 The number of research articles on single-cell metabolism in the past twenty years. Over the past two decades, research work on single-cell metabolism has been tracked according to publication dates . The number of studies related to single-cell metabolism and its methodological research has been increasing year by year.

**Figure S2**


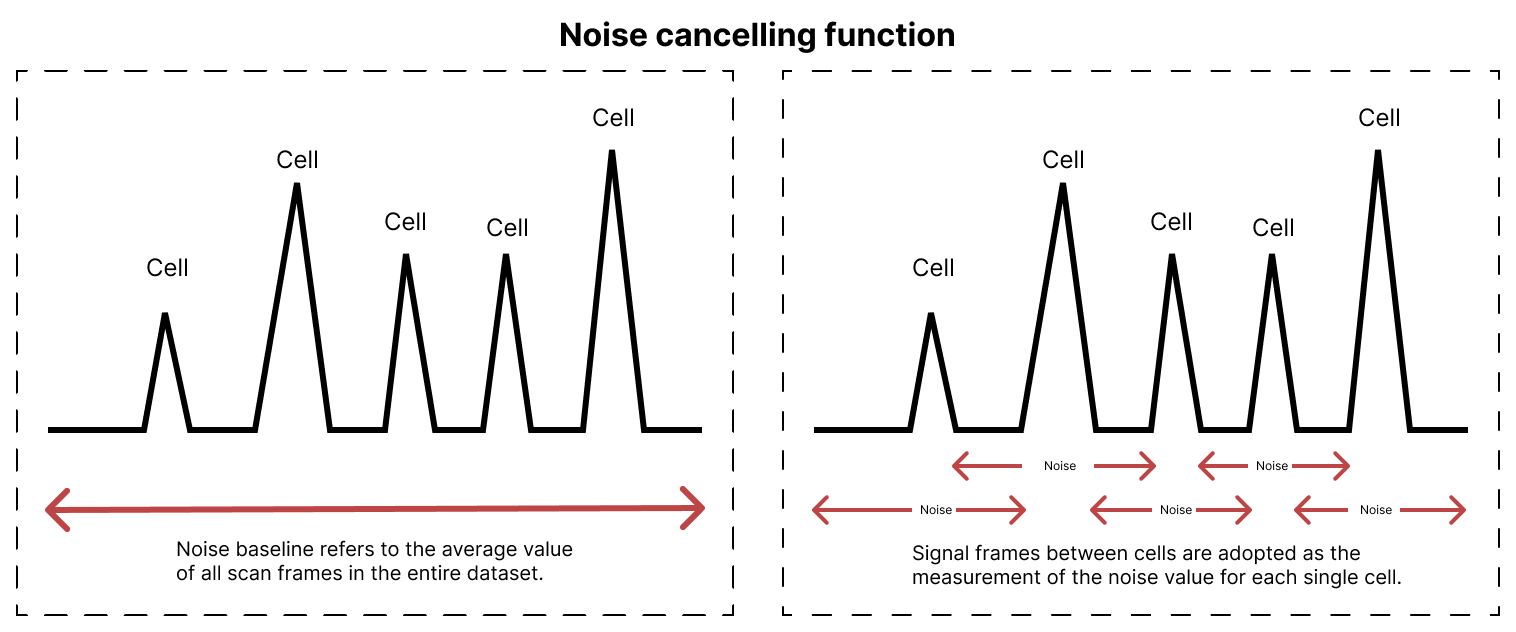


Figure S2 In terms of noise management in single-cell metabolic data, rather than resorting to a global mean calculation, we’ve implemented a method akin to rolling average, allowing for a cell-specific blank noise interval match.

**Figure S3**


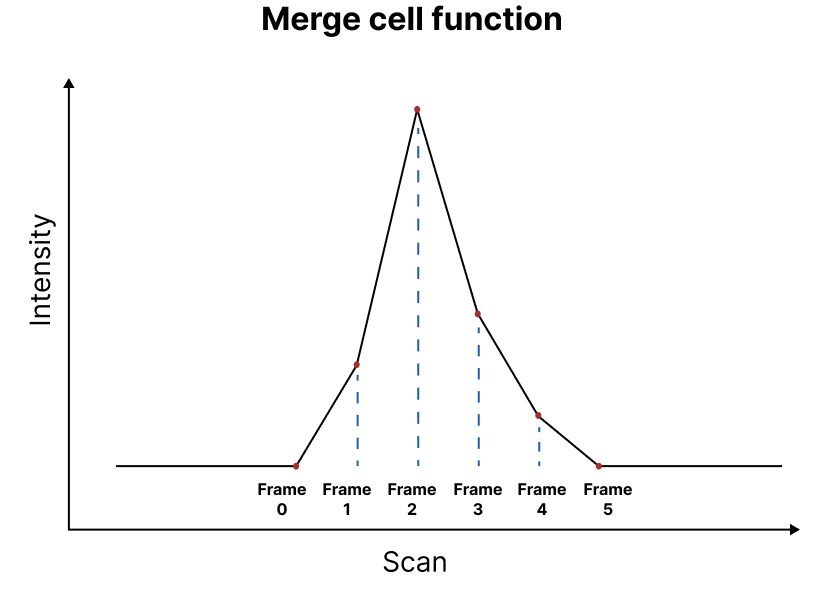


Figure S3 With respect to individual cell data that’s continually detected, we’ve elected to employ a combined tactical approach to obtain a complete set of metabolic data for each cell.

**Figure S4**

Figure S4 The starting point of each cell in the data obtained using the peak finding algorithm in SCMeTA.

**Figure S5**

Figure S5 As time passes, the metabolic signals in the solution are gradually increasing.

**Figure S6**


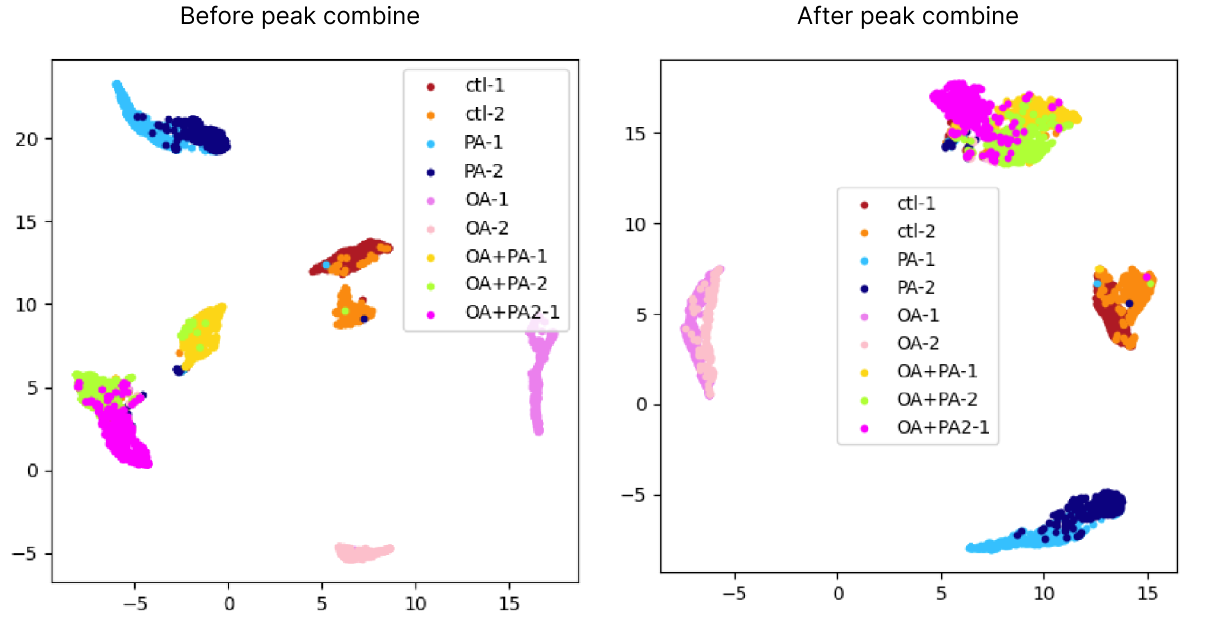


Figure S6 The UMAP classification results processed by different algorithms show that the classification results are affected by the offset of the mass spectrometry quality axis.

**Figure S7**


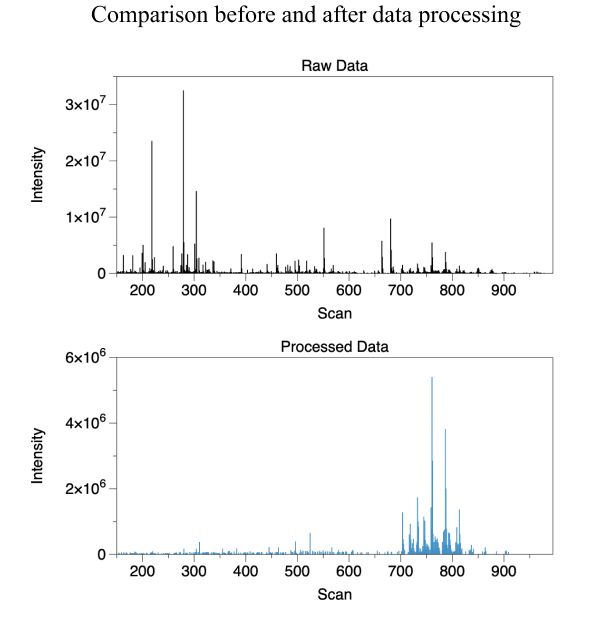


Figure S7 In droplet microextraction experiments, the raw data of the experiment is processed by SCMeTA. After processing, the noise signal in cells is significantly reduced, while lipid signals and small molecule signals are retained.

**Figure S8**


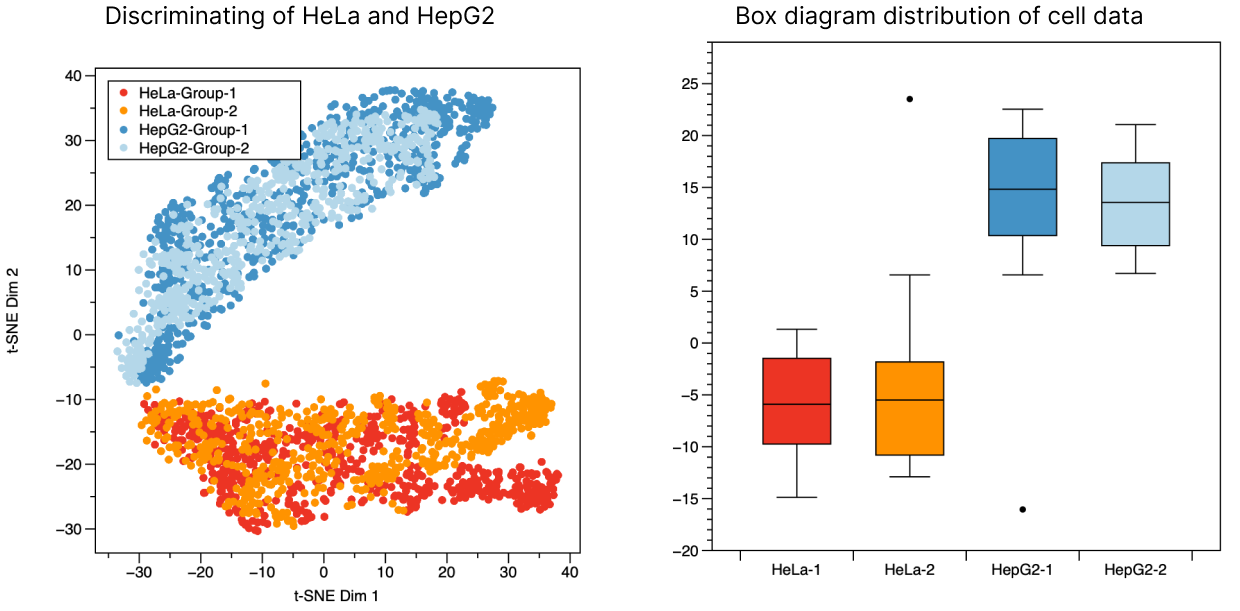


Figure S8 Using SCMeTA for data processing and classification of HeLa cells and HepG2 cells, the clustering effect is good for different cell types, and there is little variation in the performance of similar cells.

**Figure S9**


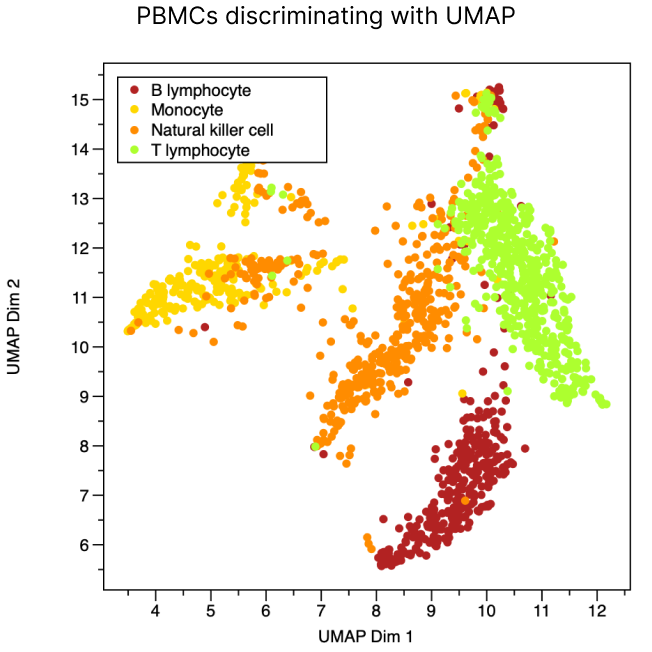


Figure S9 For data processing of human PBMCs cells, good cell differentiation results are obtained.

**Figure S10**

Figure S10 The distribution of unfiltered signals in 600 cells, with the vast majority of signals appearing less than 10% of the time.

**Figure S11**

Figure S11 The relationship between the filtering threshold ratio and the count of metabolites, highlighting a sharp decrease in the number of metabolites as the threshold approaches 0.1-0.2.

**Default process parameters**

sc = Process(refer_mz=760.58)

# Load data, path can be a dir path or a file path.

sc.load(path = 'data.RAW')

# Pre-process data

sc.pre_process(resolution: float = 0.01, count: int = 10)

# Process single-cell metabolism data.

sc.process(

max_ratio: float = 0.1

adjacent: int = 3,

snr: float = 3,

resolution: float = 0.1,

threshold: float = 0.2,

lock_mz: bool = False,

filter_method: str = "all"

)

# Post-process data

data: dict[name, SCData] = sc.post_process(

normalize_method=["log", "zscore"]

fillna_method: str = "none",

)

# Save results

sc.save()
